# *Notes from the Field:* Exposures to Mpox Among Cases in Children Aged ≤12 Years — United States, September 25–December 31, 2022

**DOI:** 10.15585/mmwr.mm7223a4

**Published:** 2023-06-09

**Authors:** Kaylea Nemechek, Ruth Stefanos, Erin L. Miller, Aspen Riser, Bethel Kebede, Romeo R. Galang, Kaitlin Hufstetler, Denisse Descamps, Adelaide Balenger, Ian Hennessee, Varsha Neelam, Helena J. Hutchins, Sarah M. Labuda, K. Meryl Davis, David W. McCormick, Grace E. Marx, Anne Kimball, Irene Ruberto, Thomas Williamson, Paul Rzucidlo, Christina Willut, Rachel E. Harold, Anil T. Mangla, Andrew English, Danucha Brikshavana, Justin Blanding, Moon Kim, Lauren E. Finn, Amy Marutani, Maura Lockwood, Shannon Johnson, Nicole Ditto, Sara Wilton, Tara Edmond, Denise Stokich, Amanda Shinall, Bryanna Alravez, Addie Crawley, Atmaram Nambiar, Emily L. Gateley, Julie Schuman, Stephen L. White, Kenneth Davis, Rania Milleron, Minerva Mendez, Vance Kawakami, Hannah E. Segaloff, William A. Bower, Sascha R. Ellington, Andrea M. McCollum, Leah Zilversmit Pao

**Affiliations:** ^1^CDC Mpox Emergency Response Team; ^2^Oak Ridge Institute for Science and Education, Oak Ridge, Tennessee; ^3^Epidemic Intelligence Service, CDC; ^4^CDC Foundation, Atlanta, Georgia; ^5^Arizona Department of Health Services; ^6^Kern County Public Health Department, Bakersfield, California; ^7^DC Department of Health, Washington, DC; ^8^Illinois Department of Public Health; ^9^Kansas Department of Health and Environment; ^10^Los Angeles County Department of Public Health, El Monte, California; ^11^Maine Center for Disease Control; ^12^Michigan Department of Health & Human Services; ^13^Missouri Department of Health and Senior Services; ^14^Jefferson County Health Department, Hillsboro, Missouri; ^15^Nevada Department of Health and Human Services; ^16^New York City Department of Health and Mental Hygiene, New York, New York; ^17^Pennsylvania Department of Health; ^18^Tennessee Department of Health; ^19^Shelby County Health Department, Memphis, Tennessee; ^20^Texas Department of State Health Services; ^21^Washington State Department of Health; ^22^Public Health – Seattle & King County, Seattle, Washington; ^23^Wisconsin Department of Health Services; ^24^Division of State and Local Readiness, Office of Readiness and Response, CDC.

During May 17–December 31, 2022, 125 probable or confirmed U.S. monkeypox (mpox)^†^ cases were reported among patients aged <18 years, including 45 (36%) in children aged ≤12 years. Eighty-three cases in persons aged <18 years diagnosed during May 17–September 24, 2022 were previously described (*1*); 28 (34%) of these were in children aged ≤12 years, 29% of whom did not have reported information on exposure. Among 20 (71%) of 28 patients with documented information on exposure, most were exposed by a household contact. This report updates the previous report using data collected during September 25–December 31, 2022, proposes possible mpox exposure routes in children aged ≤12 years, and describes three U.S. mpox cases in neonates. Household members or caregivers with mpox, including pregnant women and their health care providers, should be informed of the risk of transmission to persons aged <18 years, and strategies to protect persons aged <18 years at risk for exposure, including isolating household contacts with mpox, should be implemented immediately.

During September 25–December 31, 2022, 17 children aged ≤12 years with probable or confirmed mpox were identified through national surveillance. CDC provided a questionnaire to state and local health departments for collection of the child’s history of exposure to any person with mpox^§^ during the previous 3 weeks, exposure settings, types of contact (e.g., skin-to-skin, being held or cuddled, diaper change, or toilet use), and precautions taken by the person with mpox (e.g., practiced isolation or covered lesions). This activity was reviewed by CDC and was conducted consistent with applicable federal law and CDC policy.^¶^

Three of the 17 pediatric patients were aged ≤7 days and had likely perinatal exposures; all three neonates were non-Hispanic Black or African American (Black). Two of these three mpox cases in neonates were previously reported (*2*). Ten of the remaining children were aged 0–4 years, and four children were aged 5–12 years. Nine patients were boys, and five were girls; nine were Black, two were Hispanic or Latino, and three were non-Hispanic White. Seven of the children aged 0–4 years, and two of those aged 5–12 years had known exposure to a person with mpox (Table); in five cases, the exposure source was unknown. Six of the seven children aged 0–4 years and both children aged 5–12 years known to be exposed to mpox were exposed by a caregiver or a household contact. Five of nine children with known exposure to a person with mpox were reported to have had close physical contact; notably, four of five children aged 0–4 years had skin-to-skin contact. Five of the nine children were exposed to a person with mpox who reported taking at least one precaution, including four persons who reported isolating. Two of these household contacts reported sharing a bed, bedroom, or bathroom with the child. Evidence suggests persons with mpox might transmit the virus up to 4 days before symptom onset (*3*); review of children’s case histories suggest that in at least three cases, the person with mpox (i.e., the exposure source) did not begin isolating until after had they received a diagnosis.

**TABLE:** Demographic characteristics and mpox exposure sources of children aged ≤12 years with mpox — United States, September 25–December 31, 2022*

Characteristic	Age group, yrs, no./No. (%)		
	All ≤12 (N = 14)	0–4* (n = 10)	5–12 (n = 4)
**Race and ethnicity^†^**			
Black or African American, non-Hispanic	**9/14 (64)**	7/10 (70)	2/4 (50)
White, Hispanic	**2/14 (14)**	1/10 (10)	1/4 (25)
White, non-Hispanic	**3/14 (21)**	2/10 (20)	1/4 (25)
**Exposure source** ^§^			
Caregiver or household contact^¶^	**8/14 (57)**	6/10 (60)	2/4 (50)
Nonhousehold contact**	**1/14 (7)**	1/10 (10)	0/4 (—)
Unknown contact	**5/14 (36)**	3/10 (30)	2/4 (50)
**Types of contact with known exposure source^††^**			
Blood or other body fluid	**0/4 (—)**	0/3 (—)	0/1 (—)
Diaper change or toilet use	**1/5 (20)**	1/3 (33)	0/2 (—)
Face-to-face	**6/7 (86)**	4/5 (80)	2/2 (100)
Feeding	**1/5 (20)**	1/3 (33)	0/2 (—)
Holding or cuddling	**3/4 (75)**	2/3 (67)	1/1 (100)
Medical care	**0/5 (—)**	0/3 (—)	0/2 (—)
Pet	**0/6 (—)**	0/4 (—)	0/2 (—)
Shared clothes, towels, bedding, or bed linens	**1/4 (25)**	1/3 (33)	0/1 (—)
Shared food or dishes	**3/4 (75)**	1/2 (50)	2/2 (100)
Shared living space	**3/6 (50)**	2/4 (50)	1/2 (50)
Shared toiletries	**1/3 (33)**	1/2 (50)	0/1 (—)
Shared toys	**0/4 (—)**	0/3 (—)	0/1 (—)
Skin-to-skin	**5/6 (83)**	4/5 (80)	1/1 (100)
**Precaution taken by known exposure source^§§^**			
Covered lesions	**3/7 (43)**	2/5 (40)	1/2 (50)
Isolated	**4/8 (50)**	3/7 (43)	1/1 (100)
Refrained from sharing	**1/8 (12)**	0/6 (—)	1/2 (50)
Wore gloves or other protective gear	**1/6 (17)**	0/4 (—)	1/2 (50)
Wore mask	**1/7 (14)**	1/5 (20)	0/2 (—)
Other	**0/7 (—)**	0/6 (—)	0/1 (—)

**Abbreviation**: mpox = monkeypox.

* Information for children aged ≤12 years in this table excludes the three cases among neonates.

^†^ Persons of Hispanic or Latino (Hispanic) origin might be of any race but are categorized as Hispanic; all racial groups are non-Hispanic. Other race responses included Asian, American Indian or Alaska Native, Native Hawaiian or other Pacific Islander, Other, and Unknown; these groups were not included because they were not identified in this group or in any cases.

^§^ Exposure to a person with confirmed or suspected mpox in the 3 weeks preceding symptom onset in the child.

^¶^ Eight household contact exposures were identified (four were direct caregiver exposures, and four were both household contacts and direct caregivers).

** One mpox exposure source was identified as being with a close friend or acquaintance during which the child’s exposure did not occur within the household.

^††^ Percentages were calculated using nonmissing data including "Yes" and "No" responses. Types of contact reported by persons with known exposure were determined by asking respondents to specify exposure type for any of the exposure settings reported. Responses were not mutually exclusive. One respondent reported two known exposures.

^§§^ Percentages were calculated using nonmissing data including “Yes” and “No” responses. Types of precautions were determined by asking respondents whether any of the precautions presented in the question were taken by the primary exposure source to prevent the spread of infection. Responses were not mutually exclusive. One respondent reported two known exposures.

This report includes the first three U.S. mpox cases reported in newborns in the 2022 outbreak; these cases were not included in the earlier report of cases of diagnosed persons aged <18 years during May 17–September 24, 2022, (*1*) but two of the cases were included in a subsequent report (*2*). Among children aged ≤12 years with mpox who had a known exposure to a person with mpox, the majority were exposed by household contacts or caregivers with mpox, consistent with previous findings (*1*). This report describes precautions taken by persons with mpox to whom pediatric patients were exposed. Not all persons with mpox reported taking precautions, and children might have been exposed to mpox before initiation of precautions. As described in a recent case report of mpox in a toddler (*4*), precautions taken might not have been sufficient to prevent transmission from caregivers to children. 

The findings in this report are subject to at least four limitations. First, because timing of initiation of precautions relative to exposure was not collected, and the number of children and infants who did not acquire mpox after exposure is unknown, effectiveness of specific precautions could not be evaluated. Second, because pediatric infections during the 2022 outbreak were rare, the sample size is small, and generalizability is limited. Third, it is not possible to investigate racial disparity of mpox cases among children and in adults because of the small number of mpox cases among children. Finally, for some cases, exposure histories might be affected by misclassification because of recall error or social desirability bias.

This report adds to the information about mpox among children during the 2022 outbreak (*5*). Early diagnosis and implementation of infection control measures are critical to reducing transmission of mpox to children and infants. Household members or caregivers with mpox, including pregnant women and their health care providers, should be informed of transmission risks to children and infants. Protecting children at risk for mpox exposure requires that exposure prevention strategies be implemented without delay. These strategies include isolating household contacts with mpox, preventing contact among adults and children with mpox and other household members, ensuring persons with mpox aged >2 years wear a mask when possible, and limiting the number of persons caring for a child with an mpox infection. When caring for the child with mpox, direct contact with the child’s rash should be avoided and gloves should be worn. In addition, postexposure prophylaxis should be considered for all members of the household.

## Footnotes

* These authors contributed equally to this report.

†
https://www.cdc.gov/poxvirus/mpox/clinicians/case-definition.html

§ Exposure to a person with mpox was defined as exposure to a person with confirmed or suspected mpox during the 3 weeks before symptom onset (e.g., any interaction with someone with mpox, a mpox-like illness, or an unexplained rash).

¶ 45 C.F.R. part 46.102(l)(2), 21 C.F.R. part 56; 42 U.S.C. Sect. 241(d); 5 U.S.C. Sect. 552a; 44 U.S.C. Sect. 3501 et seq.
